# Basophil response to peanut allergens in the mediterranean area

**DOI:** 10.1186/2045-7022-3-S3-P167

**Published:** 2013-07-25

**Authors:** C Mayorga, MJ Torres, AB Blazquez, F Gomez, A Aranda, S Koppelman, A Diaz Perales, S Vieths, M Blanca

**Affiliations:** 1 Resarch Laboratory, IMABIS Foundation-Carlos Haya Hospital/Bionand, Malaga, Spain; 2 Allergy Service, Carlos Haya Hospital, Malaga, Spain; 3 HAL Allergy, HAL Allergy, Leiden, the Netherlands; 4 Centre for Plant Biotechnology and Genomics, UPM-INIA, Pozuelo de Alarcon, Spain; 5 Division of Allergology, Paul-Ehrlich-Institut, Federal Institute for Vaccines and Biomedicines, Landen, Germany

## Background

The major peanut allergens Arah1, Arah2, and Arah3 are considered the more important sensitizers in peanut allergy, although the lipid transfer protein (LTP) Arah9 has been shown relevant in Mediterranean areas. We evaluated the basophil activation response (BAT) to different peanut allergens and the peach LTP Prup3 in patients with peanut allergy.

## Methods

We included 60 subjects in different groups: Group 1, peanut and peach allergy; Group 2, peanut allergy and good tolerance to peach; Group 3, peach allergy and good tolerance to peanut; Group 4, good tolerance to peach and peanut. The diagnostic was performed by clinical history, skin prick test and/or double blind placebo controlled food challenge. BAT to Arah1, Arah2, Arah3, Arah6, rArah9 and rPrup3 was done using anti-IgE and CD63 and cells analyzed in a FACSCalibur flow cytometer.

## Results

Comparisons of BAT results between peanut allergic patients (Group 1 and 2) and controls (Group 4) showed increased basophil activation in Group 1 with Arah2 (p=0.031) and Prup3 (p=0.009) whereas Group 2 had an increase with Arah1 (p=0.016), Arah2 (p=0.001) and Arah9 (p=0.016), but not with Prup3. Comparisons with subjects allergic to peach with good tolerance to peanut (Group 3) showed a BAT increase only with Arah2 (p=0.009) in Group 2. Finally, the comparisons between groups with good tolerance to peanut (Groups 3 and 4) showed significant differences for peach allergic patients with Arah9 (p=0.004) and Prup3 (p=0.008).

## Conclusion

The peanut allergen Arah2 is the best to discriminate peanut allergy in a Mediterranean population where LTP is highly prevalent.

## Disclosure of interest

None declared.

